# Ameliorative effects of sea buckthorn oil on DNCB induced atopic dermatitis model mice via regulation the balance of Th1/Th2

**DOI:** 10.1186/s12906-020-02997-2

**Published:** 2020-08-26

**Authors:** Xinxin Wang, Sijia Li, Jiping Liu, Dongning Kong, Xiaowei Han, Ping Lei, Ming Xu, Hongquan Guan, Diandong Hou

**Affiliations:** 1 Liaoning University of Traditional Chinese Medicine, Shenyang, Liaoning PR China; 2 Basic Medical and Forensic Medicine, Baotou Medical college, Baotou, Inner Mongolia PR China; 3 Neurosurgery Department, Northern Hospital of Inner Mongolia, Baotou, Inner Mongolia PR China; 4 Liaoning Dongning Pharmceutical Co., Ltd., Fuxin, Liaoning PR China; 5 College of Integrated Traditional Chinese and Western Medicine, Liaoning University of Traditional Chinese Medicine, Chongshan Road NO.79, Shenyang, Liaoning 110847 P.R. China

**Keywords:** Atopic dermatitis, Sea buckthorn oil, 2,4-dinitrochlorobenzene, Cytokine

## Abstract

**Background:**

Atopic dermatitis (AD) is a worldwide chronic skin disease which burden public health. Sea buckthorn (SBT) (*Hippophae rhamnoides* L., Elaeagnaceae) oil, as a traditional herbal medicine, has been used for disease treatment for many years. The effects of SBT oil on AD mouse model induced by repeated administration of 2,4-dinitrochlorobenzene (DNCB) in BALB/c mice was evaluated in this study.

**Methods:**

Mice were divided into four groups including the normal control group, AD model group, AD model group treated with SBT oil (5 ml/kg) and AD model group treated with SBT oil (10 ml/kg). Same volume at different concentrations of SBT oil was applied daily on the latter two groups by gavage for 15 days following AD model induction. The function of skin barrier and the production of IL-4, IFN-γ, TNF-α and TSLP were examined after animal sacrifice. The migration and mature of langerhans cell (LCs) in lymph node was further assessed by flow cytometry.

**Results:**

SBT oil alleviated dermatitis scores, decreased ear thickness, prevented infiltration of mast cell, reduced lymph node weight and depressed activity of Th2 cells. SBT oil also reduced the expression of IL-4, IFN-γ, TNF-α and TSLP in ear tissue, IgE level in serum and mRNA relative expression of IL-4, IFN-γ, TNF-α in lymph node. Moreover, SBT oil inhibited the migration of LCs cells from local lesions to lymph node and it’s mature in lymph node.

**Conclusions:**

These results suggest SBT oil had a beneficial effect either systemic or regional on DNCB-induced AD mice via maintain the balance of Th1/Th2 and may be a potential complementary candidate for AD treatment.

## Background

AD is a chronic inflammatory skin disease characterized with eczematous pruritic rash which has high morbidity in children and could be recurrent in adulthood [1, 2]. As a general public health disease, the prevalence of AD has increased in recent years [3, 4]. AD affects nearly 20% of children and 3% of adults worldwide and the incidents become higher and higher [5]. Although the pathogenesis of AD is not explicit utterly, genetic risk, environmental factors, skin barrier dysfunction and immune dysregulation are thought to play important roles during the pathogenesis of AD [5–8]. As for immune dysregulation, Th2 skewing seems to be the key point of AD pathogenesis [9, 10]. Immunological disorder of Th1/Th2 balance due to strong type 2 immune responses characterized by over infiltration of mast cell, increased production of Th2 cytokines and IgE level in serum plays crucial role in the onset and process of AD. These Th2 cytokines subsequently induce the release of other proinflammatory cytokines such as IFN-γ through activating of Th1 cells [11].

Due to the heavy burden AD placed on society and patients [12, 13], treatment approaches are needed imperatively. In clinical practice, regional emollient and systemic corticosteroids were generally used to cure AD [14, 15]. However, experts of International Eczema Council reached on a conclusion that although the use of corticosteroids for AD is widespread, it is also discouraged due to the side effects and the risk of rebound. In consideration of potential fearful side effect of topical steroid and immunosuppressive application [14, 16], there is a strong enthusiasm in seeking alternative and complementary medication to treat AD. Recently, seeking new potential candidate from natural materials for AD management attracted greatly attention [11, 17–20].

SBT is a wild deciduous shrub or dwarf tree belonging to the Elaeagnaceae family which has been used in Tibetan, Mongolian and Chinese traditional medicine extensively for disease management [21–23]. According to many researchers SBT has various medicinal effects such as anti-tumor, anti-stress, anti-inflammatory, anti-ulcer, anti-oxidant, healing, regulation of cardiovascular and immune system [24–28]. SBT oil which contains rich fatty acids, tocopherols, ω3 and ω6 etc. is a main bioactive part of SBT and it has been proved to have beneficial effect on skin inflammation conditions and have the ability to improve the composition of fatty acid in skin [29, 30]. Therefore, this study carried out to explore the beneficial effects of SBT oil on DNCB-induced AD mouse model and its possible mechanism.

## Methods

### SBT oil

SBT oil was provided by Liaoning Dongning Pharmceutical Co. LTD. The oil was extracted from the dried press residue (including berry flesh, seeds and peel) of SBT juice processing by aseptic supercritical carbon dioxide process. Analysis of samples was performed using a HP-5MS capillary column (30 m × 0.25 mm, 0.25 μm, Agilent technologies Inc., Santa Clara, CA, USA) in a GC/MS (5975C, Agilent technologies Inc). Sample was injected into the column and ran using split mode (split ratio = 10:1). The helium carrier gas was programmed to maintain a constant flow rate of 1 ml/min. Oven was initially 80 °C for 3 min, then finally raised to 300 °C at 4 °C/min. Fatty acids were identified by a reference standard mixture FAME (Supelco, Bellefonte, PA, USA) analyzed under the same operating conditions as those employed for FAME of the samples. The components in SBT oil are exhibited in Table 1.

**Table 1:** Major fatty acids and contents of sitosterol and β-carotene in SBT oil

Fatty acids (%)							Sitosterol (mg/g)	β-carotene (mg/g)
Myristic acid (C14:0)	Palmitic acid (C16:0)	Palmitoleic acid (C16:1)	Stearic acid (C18:0)	Oleic acid (C18:1)	Linoleic acid (C18:2)	Linolenic acid (C18:3)		
0.83	26.87	27.52	1.2	33.1	4.3	1.5	15.0	6.9

### Animals and animal treatment

**Fig. 1:**
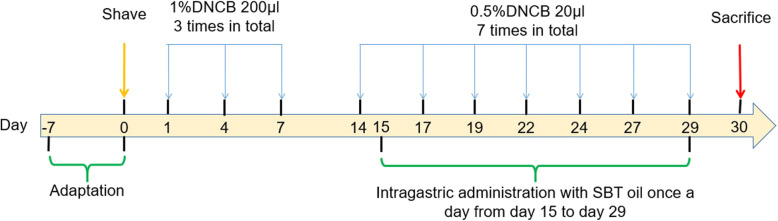
General schematic diagram for AD model induction and SBT oil treatment

Female healthy specific pathogen-free BALB/c mice, aged 6-8 weeks, weighted 20 ± 2 g, were provided by Liaoning Changsheng Biotechnology Co., Ltd. (Benxi, China). All mice were housed in groups of 6 mice per cage waiting to be grouped in a specific pathogen-free environment in 12 h light-dark cycle and allowed free to water and food. Mice were acclimatized for 1 week before AD model induction. Mice were randomly divided into 4 groups: the normal control group, AD model group, AD model group treated with SBT oil (5 ml/kg) and AD model group treated with SBT oil (10 ml/kg), each group with 6 mice. The dorsal skin of each mouse was shaved following DNCB (200 μl,1%) sensitization three times in total from day 1 to day 7 and the skin of left ear was challenged by DNCB (20 μl,0.5%) seven times in total from day 14 to day 29. AD model group treated with SBT oil (5 ml/kg) was given 0.1 ml SBT oil plus 0.1 ml olive oil per mice, AD model group treated with SBT oil (10 ml/kg) was given 0.2 ml SBT oil per mice. Oil was applied by intragastric administration once a day from day15 to day 29, olive oil (0.2 ml) was given for the normal control group and AD model group at the same time. At the same time the thickness of left ear was measured every 3 days. All animals were sacrificed at day 30 and samples including blood, left ear tissues and submaxillary lymph nodes were collected, the mice were anesthetized with 1.2% avertin solution (0.5 g 2,2,2-tribromoethanol powder dissolved into 1 ml 2-methyl-2-butanol and 39 ml PBS at 55 °C) which was filtered using a Nalgene 0.22 μm filter (Thermo Fisher Scientific, Inc., Waltham, MA, USA) and sacrificed via exsanguination [31]. The full-scale procedures of AD model induction and treatment are shown in Fig. 1. All experimental procedures performed were approved by the Ethical Committee of Experimental Animal Care at Liaoning University of Traditional Chinese Medicine (Shenyang, PR China).

### Evaluation of AD severity

Dermatitis severity was assessed by ear thickness and dermatitis scores through the method of blind. Ear thickness was measured every 3 days since day 15 with a digital thickness gauge (Mitutoyo Co., Kanagawa, Japan). Dermatitis scores was calculated according to 4 main characteristics: dryness/crusting, hemorrhage/erythema, erosion/excoriation and edema [32]. Each one was marked on a scale from 0 (none), 1 (mild), 2 (moderate), to 3 (severe). The overall dermatitis score was consist of the sum of individual scores which range from 0 to 12 [33].

### Histopathological analysis

The left ear samples of mice were collected on day 30, then fixed in 10% formalin and embeded in paraffin. The sections (5 μm thick) were stained either with hematoxylin and eosin (H&E) for visualizing dermal and epidermal thickness or with toluidine blue (TB) for visualizing mast cell numbers. The mast cells were counted in 3 sections of power fields at 200 × magnification.

### Immunohistochemistry

In short, after deparaffinization and rehydration the ear tissue slides were treated with 0.3% hydrogen peroxide-methanol for inhibiting endogenous peroxidase and with high pressure for antigen retrieval. Then the slides were incubated with sheep serum for 30 min at 37 °C, with primary antibodies (Abcam) for overnight at 4 °C, with secondary antibodies provided by Zhongshanjinqiao (Beijing, China) for 1 h at 37 °C. At last the slides were stained with diaminobenzidine (DAB) provided by Zhongshanjinqiao (Beijing, China) for coloration. Result was analyzed by ImageJ.

### Real-time polymerase chain reaction (RT-PCR)

Mouse submaxillary lymph nodes were collected and weighted while sacrifice and total RNA was extracted from lymph node tissues using TRIzol reagent (Invitrogen; Thermo Fisher Scientific, Inc.) following the manufacturer’s protocols and reverse transcribed with Prime ScriptTMRT reagent kit (TaKaRa Biotechnology Co., Ltd., Dalian, China). Real-time polymerase chain reaction analyses were performed under the protocols of SYBR®Premix Ex TaqTM II (TaKaRa Biotech Co., Ltd., Dalian, China) and the primers used in this study were designed as shown in Table 2. Relative quantities of all targets in test samples were normalized to their corresponding GAPDH levels. The 2^-ΔΔCt^ method was used to calculate relative expression quantify.

**Table 2:** Primers used for RT-PCR

Gene	Primer sequence
IL-4	Forward: 5′-ACAGGAGAAGGGACGCCAT-3′
	Reverse: 5′-GAAGCCCTACAGACGAGCTCA-3′
IFN-γ	Forward: 5′- TGAGCTCATTGAATGCTTGG −3′
	Reverse: 5′- GGCCATCAGCAACAACATAA −3′
TNF-α	Forward: 5′-GGAAAGGACGGACTGGTGTA-3′
	Reverse: 5′- TGCCACTGGTCTGTAATCCA −3′
GAPDH	Forward: 5′-TGGTGAAGGTCGGTGTGAAC-3′
	Reverse: 5′-ACTGTGCCGTTGAATTTGCC-3′

### Flow cytometry

The antibodies used for flow cytometry were provided by BD (USA) and the scheme was performed follow induction. About 1 × 10^6^–5 × 10^6^ cells of submaxillary lymph node was collected in EP tube, centrifuged at 1500 rpm and 4 °C for 5 min. One hundred μl PBS was mixed with cells after discarding the supernatant, then another 100 μl PBS containing fluorescent antibodies was added for staining at 4 °C for 20 min and the process was kept out of the sun. Five hundred μl PBS was added and blended repeatedly for washing then centrifuged at 5000 rpm and 4 °C for 5 min. The supernatant was discarded carefully and another 500 μl PBS was added and mixed. Finally, the mixture was transferred to flow tube for flow cytometry.

### Statistical analysis

The data is presented as mean ± SD. The significance of differences of different groups was evaluated by One-way analysis of variance (ANOVA) followed by the Dunnett t test. P<0.05 was considered statistically significant.

## Results

### SBT oil has a beneficial effect on skin against the development of DNCB-induced AD models in BALB/c mice

**Fig. 2:**
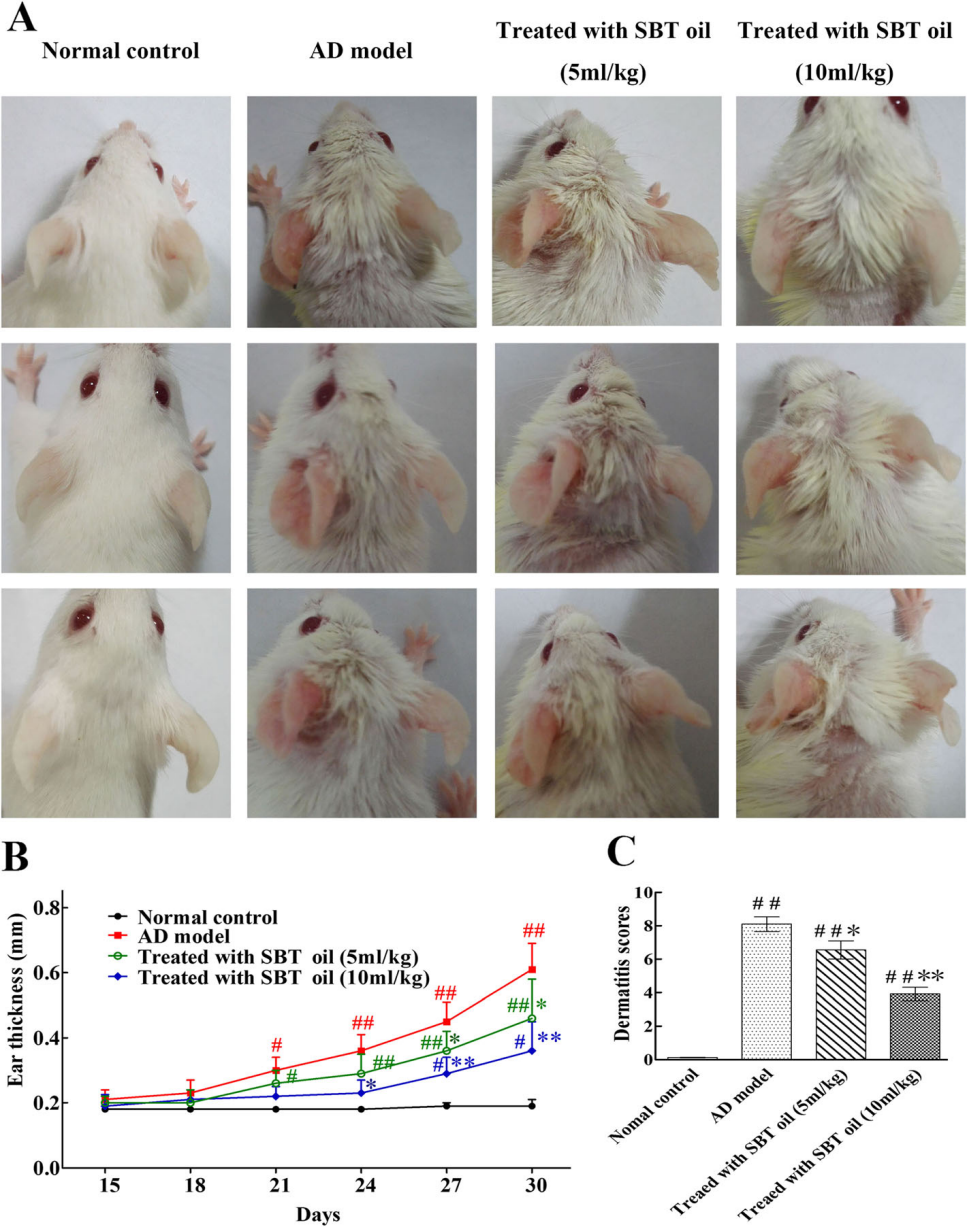
Effects of SBT oil on AD model skin induced by DNCB. **a** Examples of characteristic of AD-like skin lesions. **b** The ear thickness of the mice. **c** The dermatitis scores were summarized by the sum of scores according to various AD symtoms. ^##^ *P* < 0.01, ^#^ *P* < 0.05vs. the control group; ** *p* < 0.01, * *p* < 0.05 vs. the AD model group

To investigate the effect of SBT Oil on AD-like skin lesions in our model, SBT Oil (5 ml/kg,10 ml/kg) was applied by gastric administration once a day following the AD model induction by DNCB showed in Fig. 1. Topical application of DNCB including sensitization and challenge induced AD-like skin lesions, presenting as erythema, itching and hemorrhage companied by abnormal scratching marks and dryness. Dermatitis severity was assessed by ear thickness and dermatitis scores. Ear thickness was measured every 3 days since day 15 and the dermatitis scores was evaluated according to 4 main characteristics as described previously. After SBT Oil administration for 15 days, the ear thickness and dermatitis scores were significantly decreased in a dose-dependent manner compared to the AD model group (Fig. 2).

### SBT oil contributed to the skin barrier repair and decreased infiltration of mast cell in AD model mice induced by DNCB

**Fig. 3:**
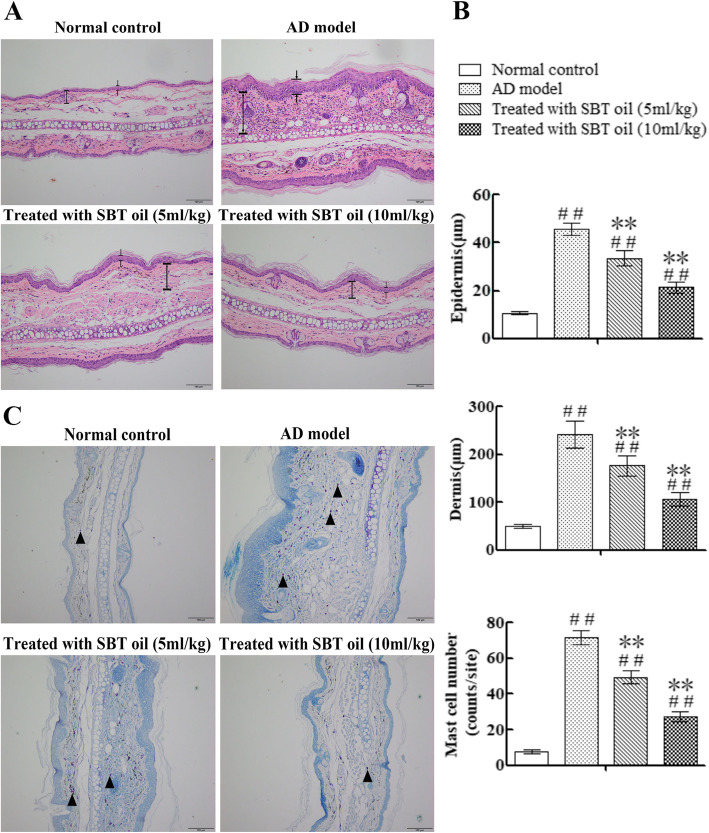
Effects of SBT Oil on histological ear skin tissue. **a** H&E staining. × 200 ( : epidermis; : dermis). **b** Dermal and epidermal thickness in H&E-stained tissue, mast cell number in TB- stained tissue. **c** TB staining. × 200( : mast cell)

To evaluate the effect of SBT oil on AD-like skin lesions histologically, H&E and TB staining were performed on tissue slides. Repetitive application of DNCB induced dermal thickening, epidermal hyperplasia and increased mast cell infiltration in AD model group. While according to H&E staining slides the dermal and epidermal thickness were both decreased and the epidermal hyperplasia was suppressed after SBT oil administration for 15 days which related to dosage (Fig. 3a, b). According to TB staining slides the infiltration numbers of mast cell were also decreased in mice treated with SBT oil (Fig. 3b, c).

### SBT oil decreased the lymph node weights in AD model mice induced by DNCB

**Fig. 4:**
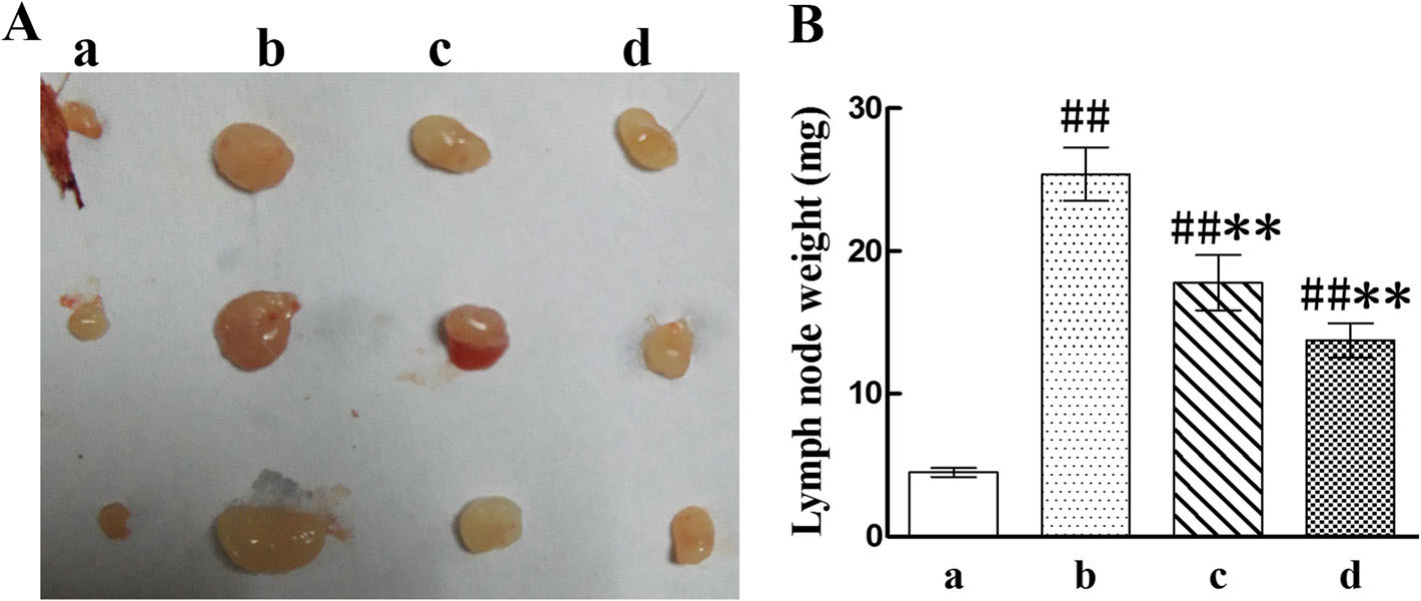
Weights of submaxillary lymph node. A submaxillary lymph node. B lymph node weight. (a). Normal control group, (b). AD model group, (c). Treated with SBT oil (5 ml/kg), (d). Treated with SBT oil (10 ml/kg). ^##^ *P* < 0.01, ^#^ *P* < 0.05 vs. the control group; ** *p* < 0.01, * *p* < 0.05 vs. the AD model group

The submaxillary lymph nodes were collected and weighted after mice sacrifice to estimate whether SBT oil play a part in the process of AD induction. The results indicated an increase in submaxillary lymph node weights in AD model group which was decreased by SBT oil administration (Fig. 4).

### SBT oil inhibited the expression of IL-4, IFN-γ, TNF-α and TSLP in ear tissue in AD model mice induced by DNCB

**Fig. 5:**
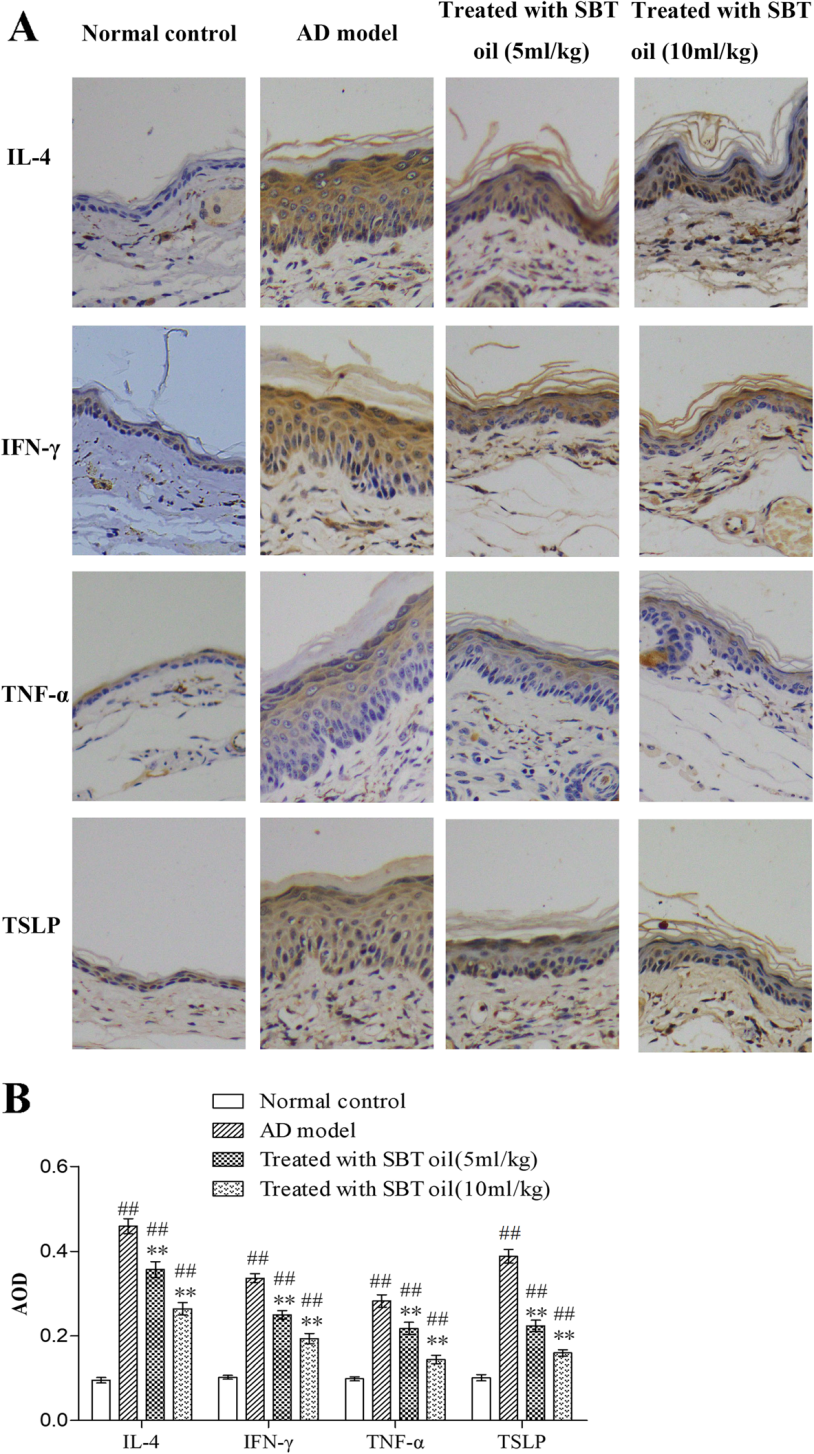
Effects of SBT oil on expression of IL-4, IFN-γ, TNF-α and TSLP in ear tissue. **a** Immunohistochemical staining of IL-4, IFN-γ, TNF-α and TSLP in ear tissue. **b** AOD analysis of IL-4, IFN-γ, TNF-α and TSLP. ^##^ *P* < 0.01 vs. the control group; ** *p* < 0.01 vs. the AD model group

In order to evaluate the effects of SBT oil on regulation of Th1/Th2 cytokines, the expression of IL-4, IFN-γ, TNF-α and TSLP in AD model mice was examined by immunohistochemistry staining on ear tissue slides. Results demonstrated an up-regulation expression of IL-4, IFN-γ, TNF-α and TSLP in AD model group which was inhibited dose-dependent by application of SBT oil (Fig. 5).

### SBT oil down-regulated IgE level in serum and mRNA relative expression of IL-4, IFN-γ and TNF-α in lymph node

**Fig. 6:**
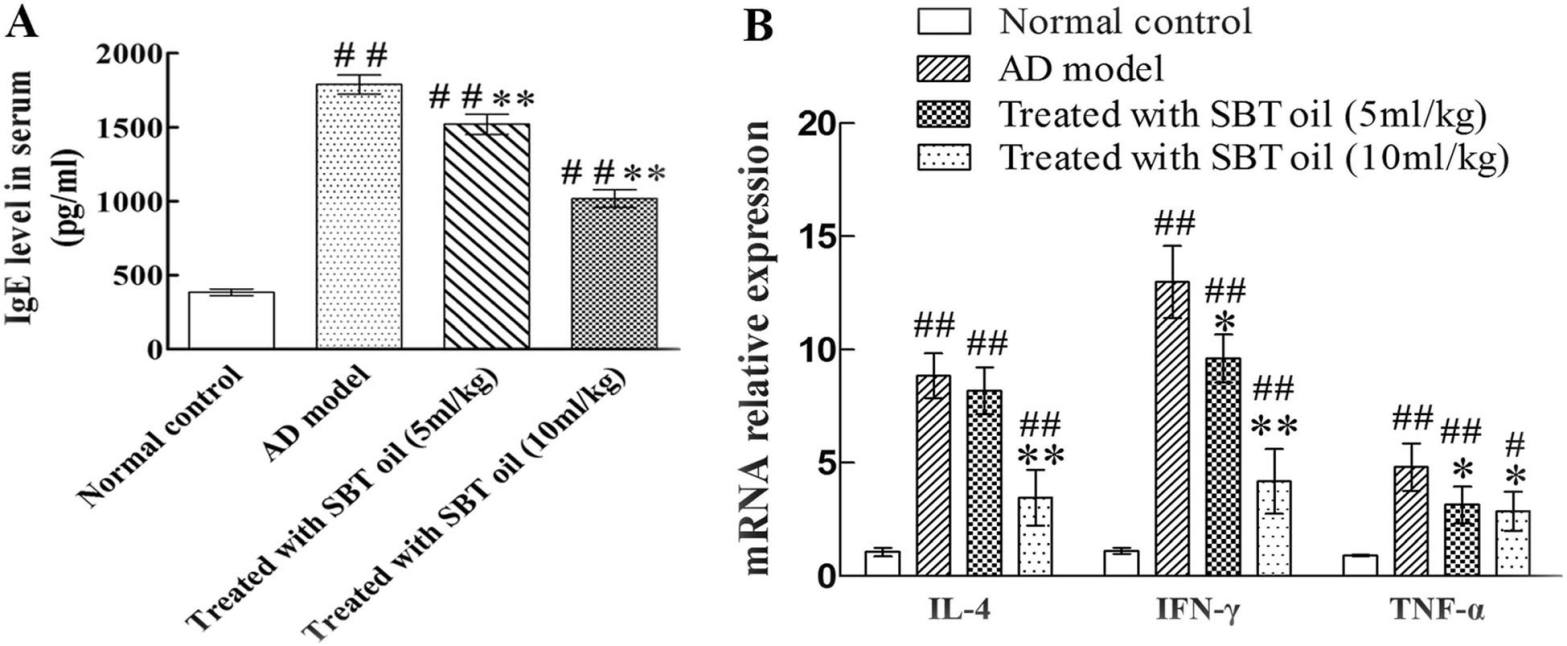
Effect of SBT oil on IgE level in serum and mRNA relative expression of IL-4, IFN-γ and TNF-αin lymph node. **a** IgE level in serum. **b** mRNA relative expression of IL-4, IFN-γ and TNF-α in lymph node. ^##^ *P* < 0.01, ^#^ *P* < 0.05 vs. the control group; ** *p* < 0.01, * *p* < 0.05 vs. the AD model group

We next measured IgE level in serum by ELISA and mRNA relative expression of IL-4, IFN-γ and TNF-α in lymph node by RT-PCR. We found that IgE level in serum was increased in AD model mice induced by DNCB. The increase was suppressed significantly in groups treated with SBT oil in a dose-dependent manner. The same as above, mRNA relative expression of IL-4, IFN-γ and TNF-α in lymph node was increased in AD model group and decreased in groups treated with SBT oil (Fig. 6).

### SBT oil decreased numbers of LCs in draining lymph node and the expressions of CD86, OX40L and MHCII on LCs

**Fig. 7:**
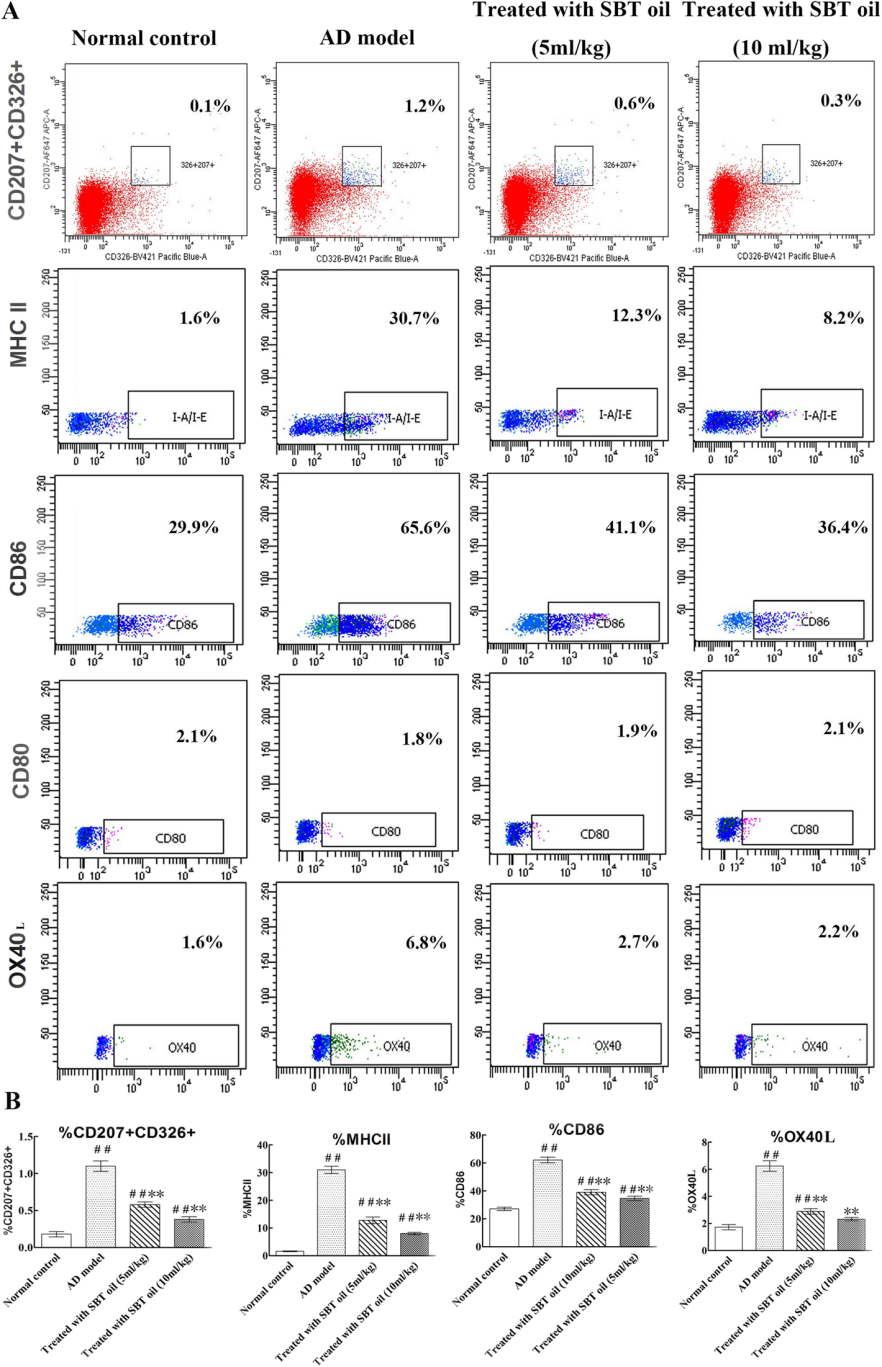
Effect of SBT oil on the maturity and migration of LC cell. **a** The scatter diagram which indicate the proportion of cells in lymph node expressing CD207^+^CD326^+^, MHC II, CD86, CD80 and OX40L. **b** The histogram of above-mentioned results. ^##^ *P* < 0.01, ^#^ *P* < 0.05vs. the control group; ** *p* < 0.01, * *p* < 0.05 vs. the AD model group

In order to assess the effect of SBT oil on the maturity and migration of LCs cell in submaxillary lymph node, we detected cell numbers expressing CD207/CD326, CD86, CD80, OX40L and MHC II by Flow Cytometry. Results as shown in Fig. 7 indicated that the expressions of CD207/CD326, CD86, OX40L and MHCIIon LCs cell in submaxillary lymph node were all increased in AD model groups induced by DNCB and decreased in groups treated with SBT oil.

## Discussion

AD is a skin inflammatory disease induced by hapten and mediated by T cells. Clinically the main characteristics of AD are erythema, edema, papule, blister, bleb, bullous reaction and even necrosis. The pathological changes of AD were infiltration of inflammatory cells and tissue edema. In this study, we employed DNCB-induced AD model using BALB/c mice which has been proposed as an appropriate representative of human AD because of similar symptoms including skin erosion, hemorrhage, epidermal hyperplasia, mast cell infiltration and increased IgE level in serum etc. SBT oil, as a traditional herbal extracts, have been proved diversified pharmacological actions such as anti-inflammatory, relieve the pressure, protecting vascular endothelial cell and immunomodulatory effects [25, 26]. The main constituents of SBT oil include Fatty acids (such as Myristic acid, Palmitic acid, Palmitoleic acid, Oleic acid, etc), Sitosterol and β-carotene. Fatty acids are crucial components of cell membranes and play important role in biological function of cells [34]. Some of the Fatty acids are required for innate immune activation and pathogen defense [35]. Sitosterol is the main constituent of many plants and vegetables and has the ability to modulate the functions of macrophages and anti-inflammation [36, 37]. β-carotene also has been shown to suppress the cellular and tissue response to inflammation [38, 39]. In view of the immunoregulation and anti-inflammatory actions of SBT oil, we assessed the anti-AD effects of SBT oil in vivo.

Topical application of DNCB followed schedule including sensitization and challenge induced AD-like skin lesions, presenting as erythema, itching and hemorrhage companied by abnormal scratching marks and dryness. The ear thickness and dermatitis scores were all significantly increased in AD model group compared to control group. After SBT Oil administration for 15 days, the ear thickness and dermatitis scores in groups treated with SBT oil were significantly decreased in a dose-dependent manner compared to the AD model group which indicate that SBT oil administration suppressed the development of AD-like skin lesions.

AD is recognized as a Th2-midiated allergic disease with over expression of Th2 cytokines and increased serum IgE level [9]. Being the antibody synthesized by plasma cells, IgE plays an essential role in some hypersensitivity, such as AD, allergic asthma and allergic rhinitis. IgE has the capability of elevating the production of Th2 cytokines. Th2 cytokines IL-4 induced immunoglobulin switching in plasma cells and resulting in up-regulation of serum IgE level. Mast cell, as one of granular leukocytes, can release many cytokines to mediate inflammatory reaction and immune regulation. These cytokines also participate in pathological manifestations of many allergic disorders including AD [40]. The infiltration of mast cell which was activated by IgE is one of the key features of AD-like skin lesions [41, 42]. Cytokines released from activated mast cells attract eosinophils into the skin which give rise to skin tissue damage. Histologically, according to TB staining slides, the numbers of mast cell infiltration in ear skin tissue of AD model mice were increased by application of DNCB and were inhibited remarkably by SBT oil. The results indicated that SBT oil has beneficial effects on suppression of skin tissue mast cell accumulation in DNCB-induced AD mice. Our TB staining results indicated that mast cells in skin tissue were scarce in control group while abundance in AD model group which highly conform to the pathological changes of AD. The mast cell number was reduced remarkably after SBT oil administration in SBT oil treated group compared with AD model group. These results suggest that SBT oil has inhibiting effect of mast cell infiltration.

According to studies published, the over expression of Th2 cytokines go hand in hand with TSLP. TSLP, which can strongly promote the differentiation of Th0 cells to Th2 phenotype through activation of dendritic cell (DCs) [43], was determined as a crucial factor in the induction of Th2 skewing in AD. The expression of TSLP has been shown to be enhanced markedly in keratinocytes of AD lesions both in AD patients and in mouse models [44]. IL-4 can in turn induce the synthesis of TSLP by keratinocytes. Importantly, the migration of DCs to draining lymph node was triggered by TSLP. More interesting, Th1 cytokines IFN-γ which can activate keratinocytes was found also elevated in AD. IFN-γ and TNF-α can synergistically stimulate the release of cytokines and chemokines in chronic stage of AD. In our study SBT oil treatment reduced the increased serum IgE level which was induced by DNCB application. Moreover, SBT oil treatment also reduced DNCB-induced increases in expression of IL-4, IFN-γ, TNF-α and TSLP in ear tissue and mRNA relative expression of IL-4, IFN-γ and TNF-α in lymph node. These results suggest that SBT oil ameliorated AD symptoms partly through the activity suppression of Th1/Th2 cells. According to the down-regulated effects SBT oil did to the TSLP expression in ear tissue, we speculated that SBT oil may have the ability to suppress both the activation and migration of DCs cell. In order to clarify our speculation, flow cytometry was used to do further study about LCs.

Draining lymph node plays an important role in the pathogenesis of AD. The weight and volume of lymph node will increase company with strengthened function when it is active. We investigated the local lymph nodes through different means. First of all, the submaxillary lymph node weights of DNCB-induced AD model mice were increased significantly which were markedly reduced after intragastric application of SBT oil for 15 days in a dose-dependent manner. We further assessed the expressions of CD207/CD326, MHC class II, CD80, CD86 on LCs and OX40L on CD4^+^ T cells in lymph node by flow cytometry because the complex immune reaction of AD was mainly taken place in lymph node. Langerin (CD207), a type II trans-membrane protein, is a C-type lection of LCs [45]. LCs are virtual mediators of immune surveillance and tolerance which resided at epidermis as DC subpopulation [46]. Antigens both external and internal were captured by LCs and presented to Th0 cells within the skin draining lymph node. CD207 is the only surface antigen just restricted to LCs [47]. Epithelial cell adhesion molecule (EpCAM,CD326), a cell surface protein, is highly expressed on LCs and appears to stimulate LCs migration [48]. Since CD207^+^CD326^+^ as the main symbol of LCs migrated into lymph node, the proportion changes showed that SBT oil suppressed the migration of LCs cell from skin lesion to draining lymph node. After degrading proteins derive from extracellular environment were taken up by endocytosis or phagocytosis and captured by MHC class II molecules, then result in peptide-loaded MHC II and migrate to the surface of antigen presenting cell waiting for recognition by CD4^+^ T cells, finally activate adaptive immune response [49]. The increased cell proportion of MHC class II indicated the uptrend tendency of mature LCs in lymph node in DNCB induced AD model mice compared with normal control mice. While this uptrend tendency was inhibited by SBT oil application in mice treated with SBT oil. Constant epidermal LCs are immature normally, and barely express co-stimulatory molecules such as CD80 and CD86. While upon LCs maturation, the expression of these co-stimulatory molecules was enhanced. In this study, SBT oil down regulated the expression of CD86 on LCs in lymph node which was enhanced by DNCB in AD model mice. It suggested the effect of SBT oil on inhibiting LCs maturation. OX40(CD134) is a transmembrane protein of tumor necrosis factor receptor super-family member which mainly expressed on activated CD4^+^ T cells and upregulated within inflammatory lesions on the antigen-activated T cells [50, 51]. TSLP can stimulate the expression of OX40 ligand (OX40L) on LCs. LCs expressing OX40L migrate from skin lesion to local lymph node and induce the transformation of Th0 cells to Th2 cells. On one hand, the increased proportion of OX40L^+^ cells in lymph node of DNCB induced AD mice was suppressed by SBT oil administration which confirmed the effect of SBT oil on activation of CD4^+^ T cells, On the other hand, SBT oil conduced to the normal function of LCs through renovating the keratinocyte and suppressing TSLP release. As a result, the abnormal Th2 skewing inflammation was inhibited by SBT oil administration.

All in all, our results suggest that SBT oil inhibited both the migration of LCs to lymph node and its maturation in lymph node, thereby inhibited the transformation of Th0 cells to Th2 cells, and finally limited the occurrence of Th2 type inflammatory response.

## Conclusion

In summary, our study results attested that SBT oil application suppressed DNCB-induced AD-like symptoms by down-regulating serum IgE level and the production of cytokines and chemokines, and regulated Th1/Th2 balance. In addition, our results also indicated that SBT oil treatment inhibited the migration of LCs to draining lymph node and its maturation. Taken together, SBT oil has excellent therapeutic effect on inflammatory skin diseases and might be a potential complementary candidate for AD treatment. In further studies, it will be worthwhile to explore the mechanism of SBT oil and its active constituent in the treatment of AD.

## Data Availability

All data and materials are contained and described within the manuscript.

## Abbreviations

AD: Atopic dermatitis
DAB: Diaminobenzidine
DCs: Dendritic cell
DNCB: 2,4-dinitrochlorobenzene
HE: Hematoxylin and eosin
IFN-γ: Interferon-γ
IgE: Immunoglobulin E
IL-4: Interleukin-4
LCs: Langerhans cell
MHCII: Major histocompatibility complex II
RT-PCR: Real-time polymerase chain reaction
SBT: Sea buckthorn
TB: Toluidine blue
Th1: T-helper 1
Th2: T-helper 2
TNF-α: Tumor necrosis factor-α
TSLP: Thymic stromal lymphopoietin
